# Fluorescent Detection of the Ubiquitous Bacterial Messenger 3′,5′ Cyclic Diguanylic Acid by Using a Small Aromatic Molecule

**DOI:** 10.3389/fmicb.2019.03163

**Published:** 2020-01-14

**Authors:** Teng-Fei Xuan, Jun Liu, Zi-Qiang Wang, Wei-Min Chen, Jing Lin

**Affiliations:** International Cooperative Laboratory of Traditional Chinese Medicine Modernization and Innovative Drug Development of Chinese Ministry of Education (MOE), College of Pharmacy, Jinan University, Guangzhou, China

**Keywords:** c-di-GMP, fluorescent, detection, quadruplex, biofilm

## Abstract

3′,5′ Cyclic diguanylic acid (c-di-GMP) has been shown to play a central role in the regulation of bacterial physiological processes such as biofilm formation and virulence production, and is regarded as a potential target for the development of anti-infective drugs. A method for the facile detection of the bacterial level of cellular c-di-GMP is required to explore the details of c-di-GMP signaling and design drugs on the basis of this pathway. Current methods of c-di-GMP detection have limited sensitivity or difficultly in probe preparation. Herein a new fluorescent probe is reported for the detection of c-di-GMP at concentrations as low as 500 nM. The probe was developed on the basis of the G-quadruplex formation of c-di-GMP induced by aromatic molecules. When used on crude bacterial cell lysates, it can effectively distinguish between the low c-di-GMP levels of bacteria in plankton and the high c-di-GMP levels in biofilm. The method described here is simple, inexpensive, sensitive, and suitable for practical applications involving the rapid detection of cellular c-di-GMP levels *in vitro* after simple bacterial lysis and filtration.

## Introduction

First discovered in 1987 (Ross et al., 1987), 3′,5′-cyclic diguanylic acid (c-di-GMP) is a ubiquitous second messenger that has been shown to play a central role in the regulation of bacterial biofilm formation (Tsiry et al., 2015). The level of c-di-GMP in bacteria has a direct impact on the formation of bacterial biofilm and is closely linked to clinical bacterial biofilm infection (Hall and Lee, 2017). Bacteria within a biofilm are several orders of magnitude more resistant to antibiotics than planktonic bacteria, and more than 80% of malignant infections are associated with bacterial biofilm resistance (Rabin et al., 2015). Consequently, targeting c-di-GMP signaling to inhibit the formation of bacterial biofilm is a new potential antibiofilm method that may overcome antibiotic resistance. As an important intracellular signal, c-di-GMP has also been confirmed to affect many other biological phenotypes of bacteria, including motility, extracellular polysaccharide, eDNA, and virulence factor production (Alina et al., 2008; Ueda and Wood, 2010; Jenal et al., 2017).

Exploration of the details of the physiological processes affected by c-di-GMP signaling is a basis for implementing regulation and drug design using this pathway. Thus, a simple and rapid method for the detection of the cellular c-di-GMP levels in different clinical strains that is suitable for practical application is urgently required to decipher more details of the relationship between c-di-GMP signaling and bacterial pathogenicity and toxicity as well as drug resistance. A readily available detection method for the determination of c-di-GMP content in bacteria is also necessary to quickly evaluate the efficacy of antibiofilm infection drugs targeting c-di-GMP. However, quantitative and selective detection of cellular c-di-GMP in complex mixed metabolites is difficult. Much effort has been invested in developing c-di-GMP detection methods, many of which can now detect c-di-GMP levels *in vivo*, but methods for quick *in vitro* overall cellular c-di-GMP level detection remain limited by poor sensitivity, complicated operation, or difficulty in probe preparation. For example, the commonly used c-di-GMP detection method is tandem HPLC-MS (Spangler et al., 2010), which offers a relatively low limit of detection but involves tedious separation and purification steps. Methods using protein-based fluorescent biosensors can be successfully applied to fluorescence imaging *in vivo* for single bacteria (Rybtke et al., 2012), but if they are used *in vitro* for quantitative detection of c-di-GMP levels, rigorous separation and extraction of the expressed proteins from bacterial cells and complicated purification to remove the prebinding of c-di-GMP are necessary. The chemiluminescent biosensor recently established by the Hammond lab for *in vitro* studies of cyclic di-GMP signaling in cell lysates (Dippel et al., 2018) also requires proteins to be expressed and purified from bacteria and minimal c-di-GMP to remain bound. RNA-based biosensor detection methods have developed rapidly in recent years; some biosensors with high sensitivity can offer real-time detection of c-di-GMP levels in living cells (Wang et al., 2016; Yeo et al., 2017, 2018), although some RNA-based biosensors have been synthesized by *in vitro* transcription for c-di-GMP detection *in vitro* (Nakayama et al., 2012; Kellenberger et al., 2015; Tsuji and Sintim, 2016), the complexity and high cost of *in vitro* transcription limit the access of detection probes in most laboratories, making it difficult for such biosensors to be widely used for *in vitro* detection. Therefore, the development of a simple yet highly sensitive *in vitro* detection method is still necessary.

C-di-GMP has been reported to form G-quadruplex in the presence of monovalent cations such as potassium ions or certain aromatic small molecular inducers, whereas other nucleic acid metabolites, such as GTP, GMP, cGMP, and c-di-AMP, lack this characteristic. Nakayama et al. demonstrated that the fluorescence of the c-di-GMP G-quadruplex inducer thiazole orange (**TO**) is enhanced in a c-di-GMP/**TO** complex (Zhang et al., 2006; Nakayama et al., 2011a,b), which can be used to establish a simple method for c-di-GMP detection *in vitro*. This method, based on an illuminating fluorescent probe, is easier to operate and the probe is easy to obtain. However, the sensitivity of the current fluorescent probe **TO** could limit the applicability of this method. The reported fluorescent probe **TO** with a detection limit of 5 μM has a relatively low sensitivity; the c-di-GMP level of bacteria is often lower than this value—typically less than 2 μM or even less than 50 nM (Hengge, 2009; Simm et al., 2009; Ho et al., 2013). Thus, a relatively large volume of bacterial culture must be processed to detect the c-di-GMP–induced fluorescent signal in the lysate. Its low sensitivity limits its application for simple and rapid detection with a large number of different bacterial samples.

A small aromatic molecule, **A18** ((E)-2-(2-(1*H*-indol-3-yl) vinyl) -3-methyl-3λ^4^-benzo [d] thiazole iodide), has been designed and synthesized. It is useful for c-di-GMP detection because of its G-quadruplex formation property and offers effective c-di-GMP G-quadruplex induction (Nakayama et al., 2011a,b; Kelsey et al., 2012), enabling the detection limit for c-di-GMP to be reduced to roughly 500 nM. **A18** can also distinguish c-di-GMP from other small nucleotides, such as GMP and cGMP. This capability is useful in *in vitro* assays of bacterial lysate samples in which other macromolecules such as DNA and RNA can be separated from the nucleotides through a simple filtration process. The improvement of sensitivity and selectivity makes the immediate detection and quantification of cellular c-di-GMP levels possible through simple lysis and filtration of bacterial samples, without the need for prior HPLC purification.

## Materials and Methods

### Materials and Instruments

Fluorescence experiments were performed on a Perkin-Elmer-LS 55 Fluorescence Spectrometer with 1 cm path length cuvette. Absorbance spectra were obtained on a ND2000C spectrophotometer with 1 cm path length cuvette. CD studies were performed by Chirascan Circular Dichroism with 1 mm path length cuvette. Synthetic materials were purchased from Bidepharm. C-di-GMP was purchased from Sigma-Innochem. **TO** was purchased from Sigma-Aldrich. rGTP, rATP, rCTP, and rUTP were purchased from Promega. dATP, dGTP, dCTP, and dTTP were purchased from Biomiga. GMP and cGMP (guanosine 3′,5′-cyclic monophosphate) were purchased from Biorbyt. ^1^H NMR spectra (Supplementary Figure S1) and ^13^C NMR spectra (Supplementary Figure S2) were recorded using a Bruker AM-400 spectrometer or a Bruker AM-300 spectrometer with DMSO-*d_6_* as the solvent and TMS as the internal standard. Electrospray Ionization-Mass Spectrometer (ESI-MS) spectra (Supplementary Figure S3) were recorded with a TRACE METM spectrometer.

### Synthesis and Characterization of Fluorescent Probe A18

#### 3-Methyl-3λ^4^-Benzo[*d*]thiazole Iodide (2)

2-Methyl benzothiazole (0.76 ml, 6 mmol) was dissolved in MeCN (10 ml). Methyl iodide (0.94 ml, 15 mmol) was added to the solution. Then, the mixture was refluxed for 24 h under N_2_. After cooling, the resulting precipitate was filtered, then washed with MeCN and Et_2_O to give a white solid 1.42 g. yield 83%. ^1^H NMR (400 MHz, DMSO- *d_6_*) δ 8.49 (d, *J* = 8.1 Hz, 1H), 8.28 (d, *J* = 8.4 Hz, 1H), 7.85 (t, *J* = 7.8 Hz, 1H), 7.77 (t, *J* = 7.7 Hz, 1H), 4.23 (s, 3H), 3.23 (s, 3H); ^13^C NMR (100 MHz, DMSO- *d_6_*) δ 177.45, 141.98, 129.70, 129.07, 128.45, 125.02, 117.26, 37.29, 18.40. ESI-MS (m/z):164[M-I]^+^.

#### (*E*)-2-(2-(1*H*-Indol-3-Yl)Vinyl)-3-Methyl-3λ^4^-Benzo[*d*]thiazole Iodide (A18)

A mixture of 2,3-dimethylbenzothiazolium iodide (150 mg, 0.52 mmol) and indole-3-carbaldehyde (90 mg, 0.62 mmol) in MeOH was refluxed for 12 h in the presence of pyridine (21 μl, 0.26 mmol). The reaction mixture was allowed to cool slowly to room temperature, and a dark solid was filtered off, washed with cold MeOH and Et_2_O, then dried to afford an orange solid **A18**, yield 74%. ^1^H NMR (300 MHz, DMSO-*d_6_*) δ 12.50 (s, 1H), 8.51-8.38 (m, 2H), 8.33 (d, *J* = 7.7 Hz, 1H), 8.29-8.23 (m, 1H), 8.13 (d, *J* = 8.3 Hz, 1H), 7.79 (t, *J* = 7.3 Hz, 1H), 7.69 (t, *J* = 7.5 Hz, 1H), 7.61-7.54 (m, 1H), 7.50 (d, J = 15.4 Hz, 1H), 7.38-7.30 (m, 2H), 4.27 (s, 3H); ^13^C NMR (75 MHz, DMSO-*d_6_*) δ 172.30, 144.64, 142.31, 138.31, 138.00, 129.27, 127.79, 127.03, 125.19, 124.36, 124.26, 122.89, 121.43, 116.34, 114.69, 113.55, 106.14, 36.16. ESI-MS (m/z):291.1[M-I]^+^.

### General Preparation of Sample Before Measurements Assay

C-di-GMP, 10 mM Tris–HCl buffer solution (pH = 7.5), and salt solutions were mixed, heated to 95°C, and kept at 95°C for 5 min, and then slowly cooled back to rt. and kept at room temperature for 10 min. **A18** was then added to the mixture and incubated in the refrigerator at −20°C overnight (about 12 h); unless otherwise stated, the reaction temperature is −20°C, the optimum temperature.

### Measurement of Fluorescence

The fluorescence instrument settings were chosen as follows: *λ*_ex_ = 485 nm (slit 6 nm), *λ*_em_ = 500–700 nm (slit 6 nm). The measurements were carried out at 15°C.

### Measurement of Ultraviolet Spectra

The UV instrument settings were chosen as follows: wavelength = 400–600 nm, temperature was 15°C. The concentration of c-di-GMP was 20 μM, **A18** was 10 μM, and buffer was 10 mM Tris–HCl (pH 7.5) containing 1 M salt (KCl, NaCl, or NH_4_Oac) or no salt was added.

### Circular Dichroism Experiments

The concentration of c-di-GMP was 70 μM, [**A18**] or [**TO**] was 30 μM, and buffer was 10 mM Tris–HCl (pH 7.5) containing 250 mM KCl. The CD instrument settings were chosen as follows: Data pitch, 1 nm; scan speed, 50 nm/min; response, 8 s; bandwidth, 1 nm.

### Preparation of Cell Lysate

PAO1*-ΔwspF* were grown up at 37°C in Luria-Bertani (LB) medium (50 ml) with static (biofilm state) or shaking (planktonic state) for 24 h, final OD (600 nm) was about 0.6, cells were pelleted by centrifugation and resuspended with 2 ml of 10 mM Tris–HCl (pH 7.5), and 200 μl of 50 mg/ml lysozyme was then added to each resuspension. The resuspension was sonicated (10/10 s, 30 min) to disrupt membranes and allow lysozyme to reach cell walls. The crude cell lysate was boiled for 10min. Each sample was centrifuged at 13,000g for 10min. The collected supernatant was then filtered by 0.2-μm filter and 3 kD exclusion columns.

## Results and Discussion

### Discovery of the New 3′,5′ Cyclic Diguanylic Acid G-Quadruplex Inducer A18

**SCHEME 1:**
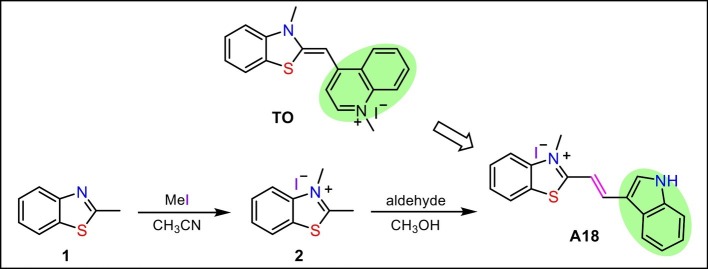
Synthetic route to the probe **A18**.

Inducing c-di-GMP to form G-quadruplex in the presence of monovalent cations and thus enhancing the fluorescence of the aromatic inducer is a promising method for the detection of cellular c-di-GMP. Because the reported inducer (**TO**) has low sensitivity and fluorescence intensity is closely related to the ability of a small molecule to induce the formation of a G-quadruplex, a new aromatic c-di-GMP G-quadruplex inducer (**A18**) that can be used as a more sensitive c-di-GMP fluorescent probe for rapid and efficient detection of cellular c-di-GMP in bacterial lysate samples was designed and synthesized in accordance with the structure of **TO**. Electron-rich S atoms are thought to be capable of interacting with the phosphate of c-di-GMP and enhancing the mutual interaction between the probe and the G-quadruplex. Thus, the benzothiazole moiety in **TO** with a positive charge was preserved as the basic nucleus in the newly developed probe. However, our experience in designing G-quadruplex ligands suggests that the introduction of groups capable of forming hydrogen bonds at the end of the molecule is extremely favorable for interaction. Accordingly, an indole group was introduced onto the benzothiazole ring to replace the quinoline part of **TO**. To expose the indole amino group, namely the hydrogen bond donor, the indole group was connected to the benzothiazole at the end of the molecule. Because aromaticity is a basic feature of an inducer, the benzothiazole and the indole were linked in a conjugated double bond to produce the new probe molecule **A18** ((E)-2-(2-(1*H*-indol-3-yl) vinyl)-3-methyl-3λ^4^ -benzo [d] thiazole iodide). This molecule was obtained using the simple two-step reaction shown in Scheme 1.

**Figure 1:**
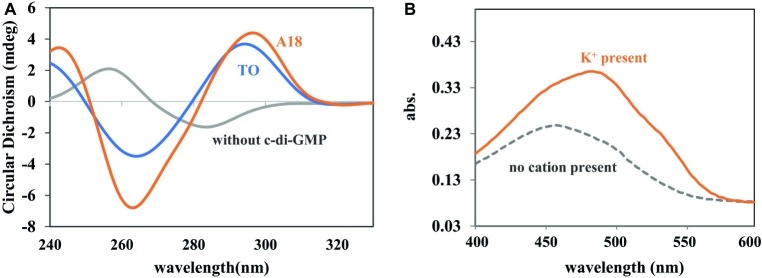
**(A)** CD spectra of c-di-GMP, c-di-GMP-**A18**, and c-di-GMP-**TO** complexes. [c-di-GMP] = 70 μM; [**A18**] or [**TO**] = 30 μM. Buffer: 10 mM Tris–HCl (pH 7.5) containing 250 mM KCl. Reaction temperature: 4°C. **(B)** UV spectra of **A18** + c-di-GMP in different buffers. Addition of K^+^ (1 M) to the buffer (10 mM Tris–HCl, pH 7.5) caused a red shift in the absorption spectrum. [c-di-GMP] = 20 μM; [**A18**] = 10 μM. Reaction temperature: 4°C.

Circular dichroism (CD) is a powerful tool with which to identify the aggregation state of c-di-GMP. When G-quadruplex is present in a solution, a positive CD peak occurs at approximately 300 nm and a negative CD peak occurs at approximately 262 nm (Zhang et al., 2006; Stelitano et al., 2013). The induction ability of synthesized **A18** was first evaluated through CD, with **TO** serving as a positive control. As shown in Figure 1A, the **A18**/c-di-GMP complex exhibited similar CD features as the reported **TO**/c-di-GMP complex, indicating that **A18** is also capable of inducing c-di-GMP to form G-quadruplex as **TO**.

### Fluorescence Enhancement Effect Upon Formation of an A18-G-Quadruplex Complex

**Figure 2:**
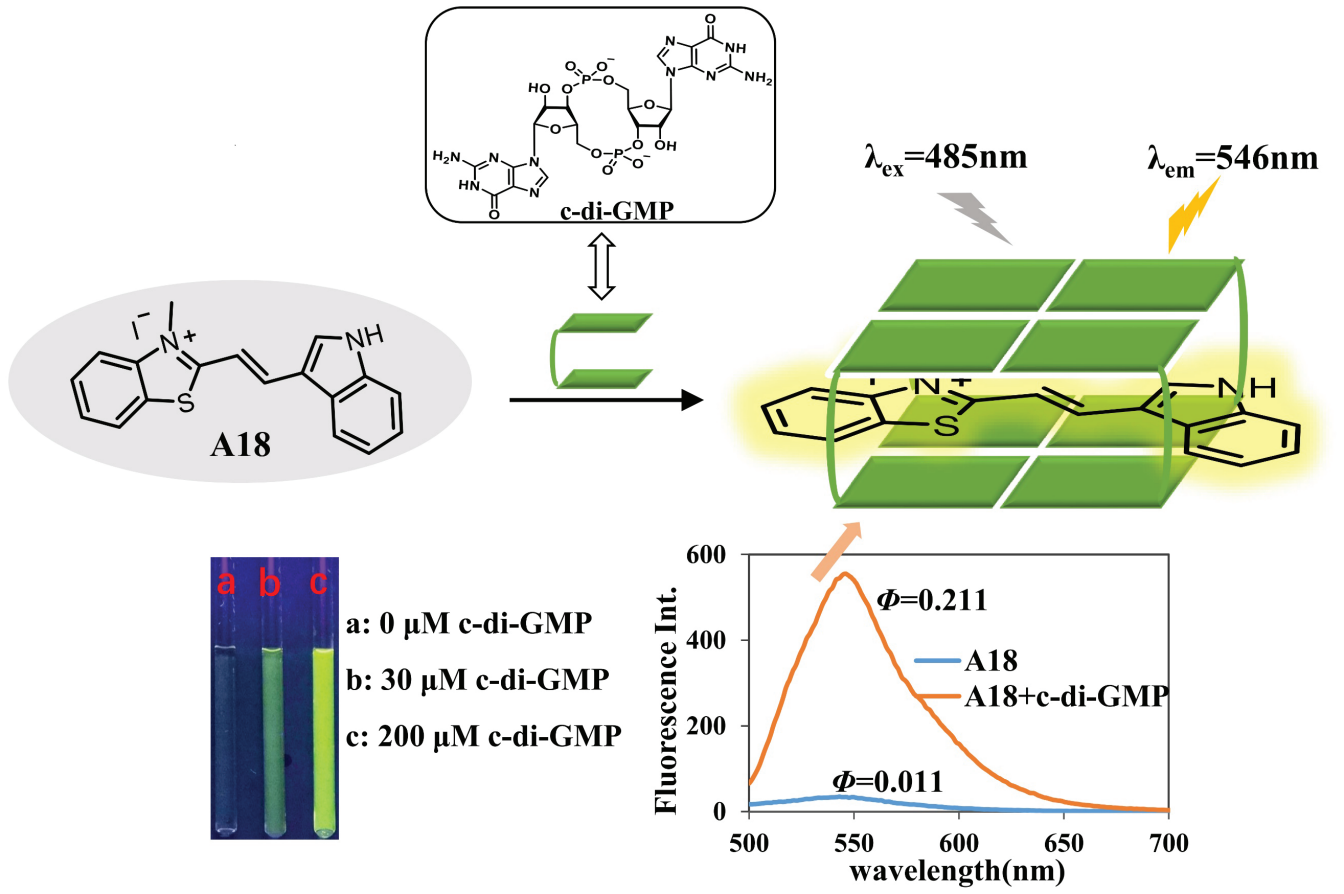
**A18** was predicted to interact with c-di-GMP to yield an **A18–**G-quadruplex complex. We hypothesized that **A18** would intercalate between the two planes of the G-quadruplex formed by c-di-GMP and that the fluorescence of **A18** would be enhanced by the restriction of its rotation.

The fluorescent molecule **A18** can bind to G-quadruplex through π–π interactions in the form of intercalation or end-stacking (Lubitz et al., 2007; Seo et al., 2008; Dutta et al., 2018), and c-di-GMP has been demonstrated to form G-quadruplex in the presence of **A18**. We hypothesized that **A18** is likely to intercalate between the two planes of the G-quadruplex formed by c-di-GMP, and because the rotation of **A18** in G-quadruplex is restricted, the nonradiative channel in excited **A18** would be closed, and the quantum yield (*Φ*) of the confined **A18** would subsequently increase, leading to fluorescence enhancement of **A18** (Figure 2). As expected, **A18** has a low fluorescence quantum yield in an aqueous solution (*Φ* = 0.011), and *Φ* increases to 0.211 when **A18** is in the confined cavities of c-di-GMP G-quadruplex complexes. This result was calculated as described in the Supporting Information using the data listed in Supplementary Table S1 (Chen et al., 2008; He et al., 2009). Further UV analysis indicated a π–π interaction between the G-quadruplex complex of c-di-GMP and **A18**, which is reflected by a red shift in the absorption spectrum when **A18** induces c-di-GMP to form the G-quadruplex in the presence of K^+^ (Figure 1B; Jin et al., 2018).

### Optimal Ion Conditions for Detection

**Figure 3:**
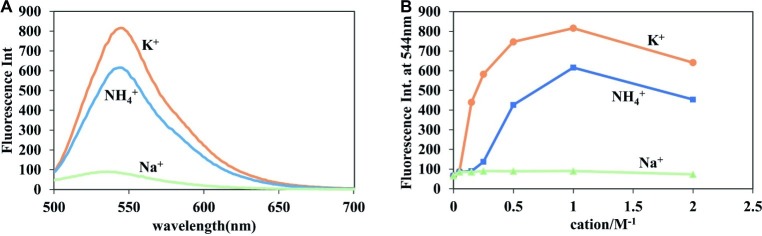
Effect of different ions on the fluorescent properties of **A18**. **(A)** Fluorescence of the **A18**-c-di-GMP complex in the presence of 1 M NaCl, KCl, and NH_4_OAc. **(B)** Fluorescence of the **A18**-c-di-GMP complex in the presence of different salt concentrations (0 mM, 50 mM, 150 mM, 250 mM, 500 mM, 1 M, and 2 M). [c-di-GMP] = 20 μM; [**A18**] = 10 μM. Ex. 485 nm, em. 500–700 nm. Buffer: 10 mM Tris–HCl (pH 7.5) containing different salts.

Because the process of fluorescence change requires the participation of the G-quadruplex, the formation of which depends on the existence of monovalent cations, the type and concentration of monovalent cations should have a large impact on fluorescence enhancement (Chen et al., 2008; Shim et al., 2009). As shown in Figure 3, the detection of c-di-GMP with **A18** was more sensitive in the presence of K^+^ than in the presence of Na^+^ or NH_4_^+^, and Na^+^ did not seem to affect the fluorescence change. UV analysis yielded similar results. In the presence of both K^+^ and NH_4_^+^, the absorption spectra exhibited a significant red shift, indicative of a π–π interaction between the c-di-GMP G-quadruplex and **A18**, but no significant change in the absorption spectra was observed when Na^+^ was present (Supplementary Figure S4). K^+^ promotes more stable G-quadruplexes (Sen and Gilbert, 1990; Olsen et al., 2006). Jones et al. demonstrated that K^+^ and NH_4_^+^ favor the formation of one or more guanine quartet complexes, whereas Na^+^ favors a bimolecular self-intercalated structure, which is consistent with our results (Zhang et al., 2006).

### Dose Response of Fluorescence on A18 and 3′,5′ Cyclic Diguanylic Acid Concentration

**Figure 4:**
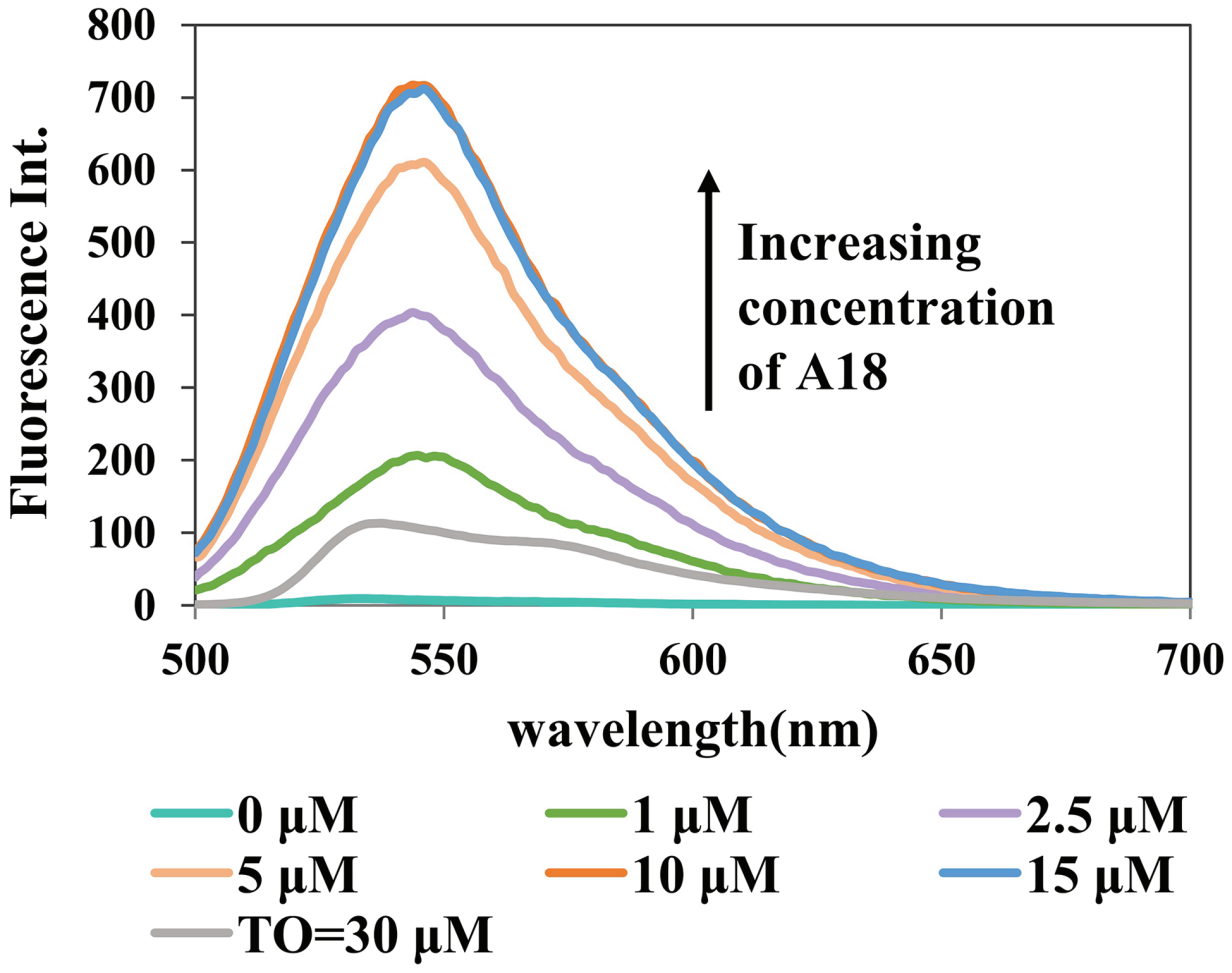
Dose response for the **A18**–c-di-GMP interaction. [c-di-GMP] = 20 μM. Buffer: 10 mM Tris–HCl (pH 7.5) containing 500 mM KCl. Ex. 485 nm, em. 500–700 nm.

**Figure 5:**
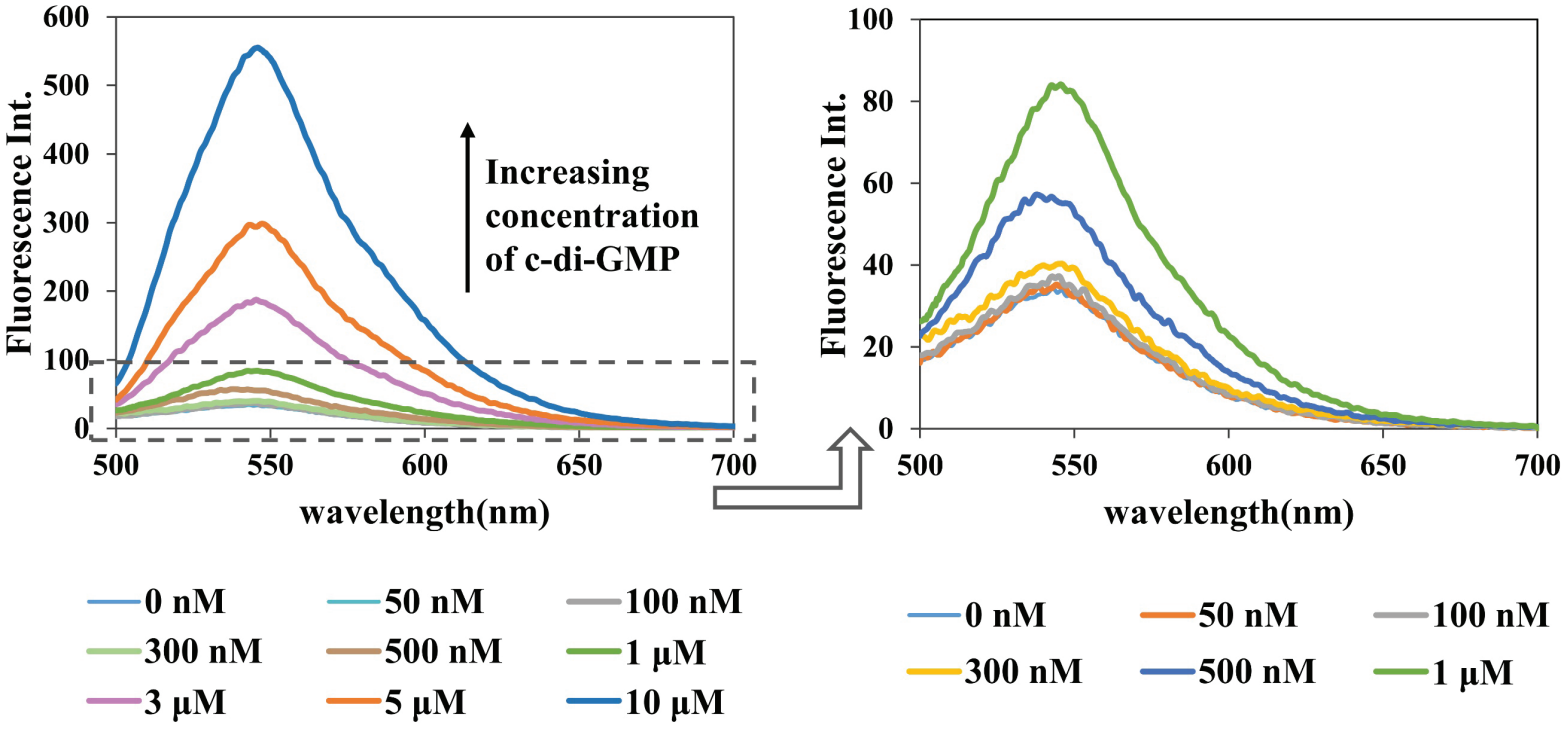
Dose response for the **A18**–c-di-GMP interaction. [**A18**] = 2.5 μM, buffer: 10 mM Tris–HCl (pH 7.5) containing 1 M KCl. Ex. 485 nm, em. 500–700 nm.

To optimize the assay conditions and investigate the detection limit of this method, the fluorescent properties of **A18** with different concentrations of c-di-GMP and **A18** were investigated using fluorescence titration. As shown in Figures 4, 5, when the concentration of c-di-GMP or **A18** increased, the fluorescence intensity was gradually enhanced. Nevertheless, obvious fluorescence enhancement was observed when the concentration of **A18** was as low as 1 μM. This is significantly stronger than the fluorescence produced by **TO** at 30 μM under the same conditions, and when the concentration of **A18** was increased to 10 μM, the fluorescence intensity was almost seven times stronger than that of 30 μM **TO** (Figure 4). These results reflect a significantly enhanced sensitivity of **A18** over **TO**. When the concentration of **A18** was >10 μM, the fluorescence intensity no longer increased (Figure 4). This indicated that **A18** has a strong ability to induce c-di-GMP aggregation and that a 10 μM concentration of **A18** may result in complete induction.

Similarly, in the presence of an increasing concentration of c-di-GMP, fluorescence intensity also increased gradually, and the detection limit for this assay was as low as 500 nM (Figure 5). Among the detection methods reported to date, using an assay to detect c-di-GMP *in vitro* with small molecules is the simplest method, but its general application is limited by its low sensitivity (Nakayama et al., 2011a,b). Furthermore, c-di-GMP, with a cellular concentration range of 0–10 μM, plays a central role in several processes that control bacterial phenotypes (Simm et al., 2009; Ho et al., 2013), and nanomolar fluorescence detection is critical to deciphering the molecular details of c-di-GMP signaling. Our research represents an advance in the detection of c-di-GMP with small molecules.

### Selective Detection of 3′,5′ Cyclic Diguanylic Acid by A18

**Figure 6:**
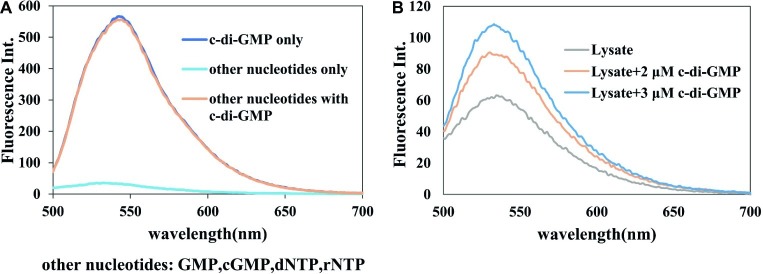
Selectivity of the detection system. Buffer: 10 mM Tris–HCl (pH7.5) containing 1 M KCl. Ex. 485 nm. em. 500–700 nm. **(A)** [c-di-GMP] = 10 μM, each [nucleotide] = 10 μM, [**A18**] = 2.5 μM. **(B)** Lysate + c-di-GMP, [**A18**] = 2.5 μM.

Having established the superiority of **A18** in the detection of c-di-GMP, we proceeded to investigate the selectivity of the method. The concentration of several other nucleotides such as cGMP, dGTP, and ATP is significantly higher than that of c-di-GMP in bacterial cells (Wang and Sintim, 2019). Therefore, it should be ascertained that the detection method can specifically detect c-di-GMP without interference from other nucleotides. It was found that c-di-GMP or a mixture containing c-di-GMP and other nucleotides (GMP, cGMP, dNTP, and rNTP) can be detected by **A18**, but a mixture of only the other nucleotides could not be detected with **A18**. The fluorescent signal of a sample that contained several other nucleotides and c-di-GMP was similar to that containing only c-di-GMP (Figure 6A). This illustrates the specificity of **A18** for fluorescence detection of c-di-GMP.

To further verify the specificity of this assay and prove the application of this assay in detection in bacterial samples, the specificity of the assay was also verified in crude bacterial lysate. As shown in Figure 6B, it can be seen that the selective detection of c-di-GMP proceeded well on crude cell lysate, without any prior separation step. After the addition of a standard sample of c-di-GMP, the magnitude of fluorescence enhancement was almost identical to that in the *in vitro* experiment, and it was apparently not affected by other components in the lysate. This also showed that our method could distinguish different concentrations of c-di-GMP in bacterial lysates, and indicates the feasibility of detection of different bacterial samples using this probe.

### Applied Research on the Detection of 3′,5′ Cyclic Diguanylic Acid in Bacterial Crude Cell Lysates

**Figure 7:**
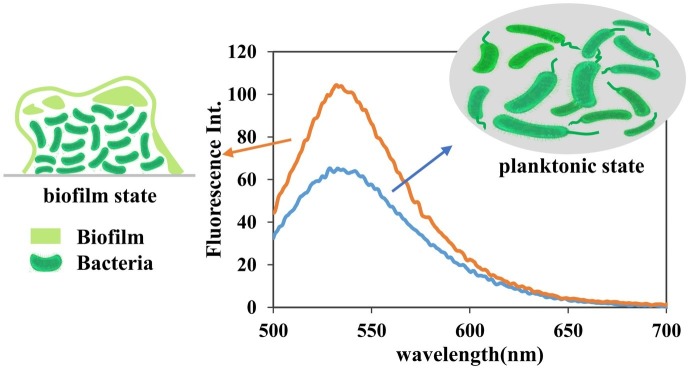
Fluorescence detection of c-di-GMP in PAO1*-Δwspf* lysate. Detection conditions: [**A18**] = 2.5 μM, buffer, 10 mM Tris–HCl (pH 7.5) containing 1 M KCl. In line with expectations, our method can reliably distinguish the differences between the two culture states even at low micromolar concentrations. Samples: 50 ml of bacterial crude lysate was concentrated to 2 ml.

The goal in this research was to develop a sensitive *in vitro* assay to quickly and easily determine the cellular levels of c-di-GMP in various bacterial samples without relying on complex steps to prepare probes or culturing large volumes of bacterial samples. The c-di-GMP detection assay using **A18** was performed on crude cell lysates with as few 50-ml bacterial samples as possible. For **TO**, 3 L of lysate was necessary. Compared with the bacteria in the planktonic state, bacteria in the static state that contributed to the formation of biofilm had a higher level of c-di-GMP (Barraud et al., 2009). Accordingly, we determined the c-di-GMP levels of PAO1*-Δwspf* (a mutant PAO1 with high expression of c-di-GMP; the construction method was as reported in Hickman et al., 2005) in planktonic and biofilm states, which have different levels of cellular c-di-GMP, by using our fluorescent probe **A18**. HPLC was also used to verify the identity of c-di-GMP and quantify the cellular c-di-GMP concentrations of the same sample (Supplementary Figure S5). We found that the peak corresponding to the c-di-GMP molecule was not obvious in the HPLC spectrum (Supplementary Figure S5), and it was difficult to quantify the c-di-GMP level even in the static state, which has high c-di-GMP expression. This may have been caused by the low level of c-di-GMP derived from the 50-ml sample and the interference of other metabolite peaks. The sample amount required for HPLC detection is generally 500 ml. Our method could distinguish well between the different c-di-GMP levels in the two culture states (Figure 7). We can infer that compared with the c-di-GMP standard addition HPLC spectrum (Supplementary Figure S5), the c-di-GMP content in the lysate was less than 1 μmol, indicating that our method can readily distinguish between the two culture states even at low micromolar concentrations. The detection method described here sensitively reflects the c-di-GMP level through fluorescence in bacterial samples, even at low micromolar concentrations, whereas HPLC and other methods are unable to detect low concentrations *in vitro* because of low sensitivity or interference from other metabolites.

## Conclusions

In summary, a new fluorescent molecule **A18** was developed for c-di-GMP detection using the property of aromatic molecule-induced G-quadruplex formation of c-di-GMP. This method is capable of detecting c-di-GMP at concentrations as low as 500 nM in a simple buffer system and has good selectivity to c-di-GMP over other small nucleotides. The method can be used for the facile and rapid detection of cellular c-di-GMP levels in bacterial lysate samples that can be obtained using a simple cell lysis and filtration process without HPLC purification. This method will contribute to the quick evaluation of the efficacy of drugs that inhibit c-di-GMP signaling as well as other explorations of this signaling.

## Data Availability Statement

All datasets generated for this study are included in the article/Supplementary Material.

## Author Contributions

T-FX, JLin, and W-MC conceived the research idea and designed the experiments. T-FX performed the experiments and wrote the manuscript. T-FX, Z-QW, JLiu, JLin, and W-MC participated in the analysis of the study and helped to revise the manuscript. All authors read and approved the final manuscript.

### Conflict of Interest

The authors declare that the research was conducted in the absence of any commercial or financial relationships that could be construed as a potential conflict of interest.
