# Supplementary material for: Fluorescent Detection of the Ubiquitous Bacterial Messenger 3′,5′ Cyclic Diguanylic Acid by Using a Small Aromatic Molecule
Source: Front Microbiol. 2020 Jan 14;10:3163. doi: 10.3389/fmicb.2019.03163 (PMC6970945; doi:10.3389/fmicb.2019.03163)
Supplement: Supplementary file 1 [file Data_Sheet_1.PDF]

Supporting Information

Fluorescent Detection of the Intracellular Ubiquitous Bacterial Messenger C-di-GMP by Using a Small Aromatic Molecule

Teng-Fei Xuan, Jun Liu, Zi-Qiang Wang, Wei-Min Chen*, Jing Lin*

International Cooperative Laboratory of Traditional Chinese Medicine Modernization and Innovative Drug Development of Chinese Ministry of Education (MOE), College of Pharmacy, Jinan University, Guangzhou, 510632, PR China

*Corresponding authors. Tel.: +86 20 8522 1367 (J. Lin), +86 20 8522 4497 (W.-M. Chen).

Fax: +86 20 8522 4766.

E-mail address: linjing_jnu@163.com (J. Lin), twmchen@jnu.edu.cn (W.-M. Chen).

Table of Contents

(1) ^{1}H NMR, ^{13}C NMR and ESI-MS spectra of fluorescent probe **A18**

(2) UV spectra of **A18** + c-di-GMP in different buffers

(3) HPLC spectra of bacteria cell lysate

(4) Calculation of fluorescence quantum yields

(1) ^{1}H NMR, ^{13}C NMR and ESI-MS spectra of fluorescent probe A18

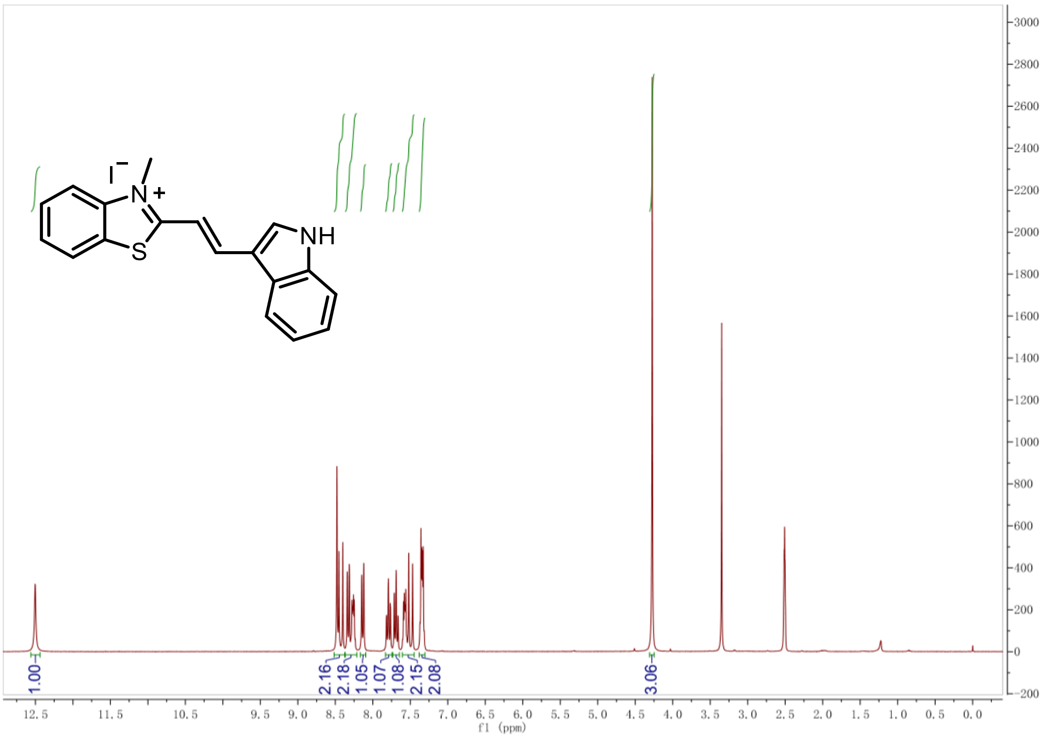

FIGURE S1. ^{1}H NHR spectra of **A18** in DMSO-d_6.

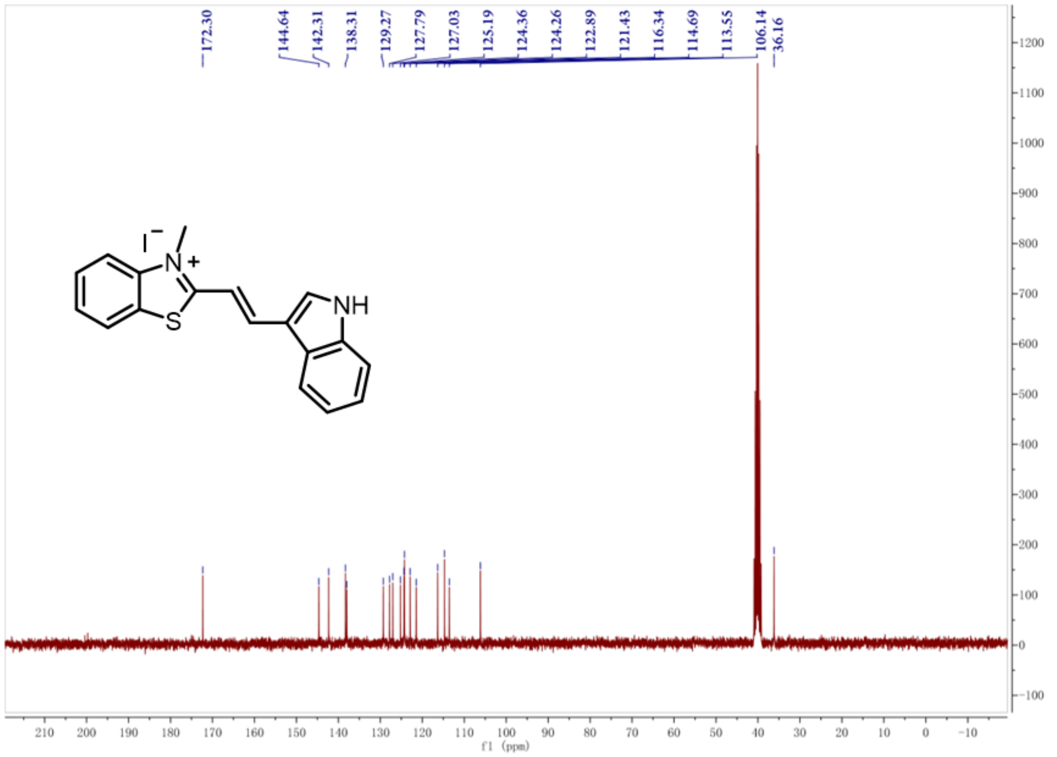

FIGURE S2. ^{13}C NHR spectra of **A18** in DMSO-d_6.

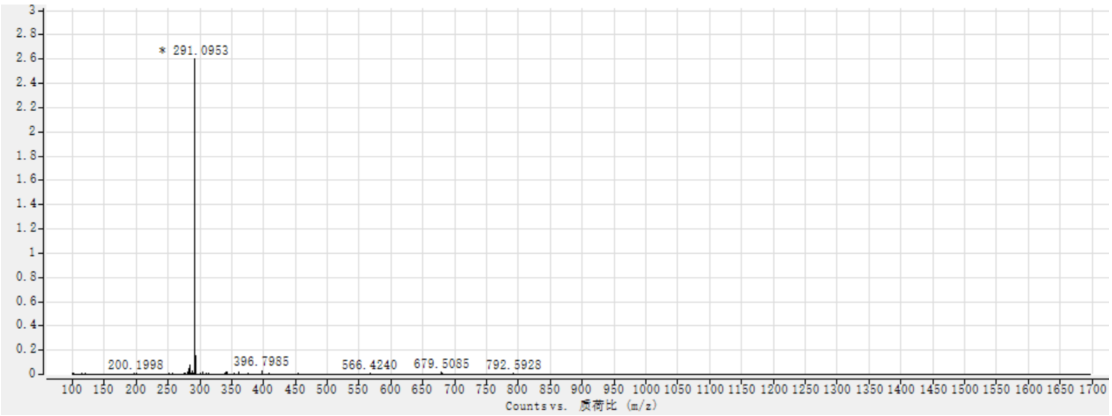

FIGURE S3. ESI-MS spectra of **A18**.

(2) UV spectra of A18 + c-di-GMP in different buffers

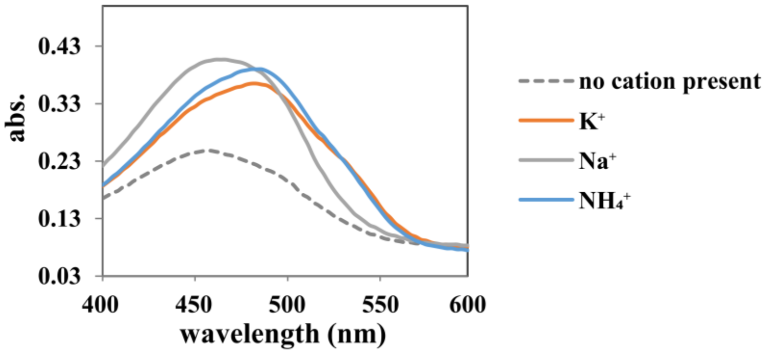

FIGURE S4. UV spectra of **A18** + c-di-GMP in different buffers. Addition of monovalent cations to **A18** causes a red shift in the absorption spectrum. Buffer: 10 mM Tris-HCl (pH 7.5) containing 1 M of one of these salts; KCl, NaCl, and NH4OAc or no salt was added. [c-di-GMP] = 20 μM, [**A18**] = 10 μM. Reaction temperature: 4 °C.

(3) HPLC spectra of bacteria cell lysate

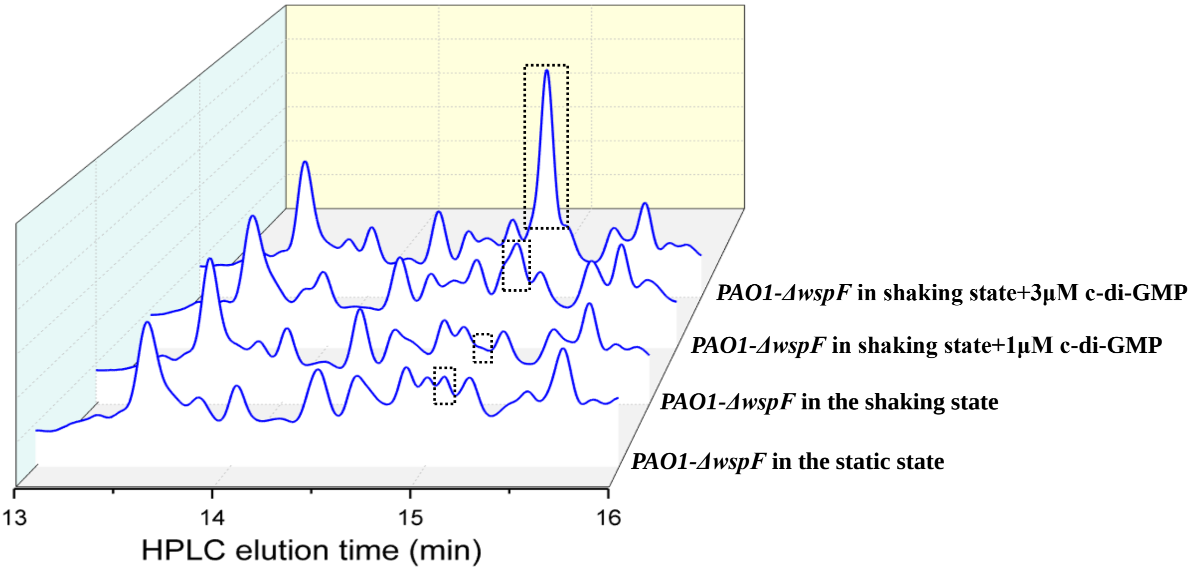

FIGURE S5. HPLC spectra of bacteria cell lysates. HPLC conditions: **(A)** 100 mM TEAA, **(B)** CH3CN, B%: 2-2%/3 min, 2-10%/15 min, 10-30%/25 min, 30-100%/30 min. Column: 5C18-MS-II. 1 ml/min. Room temperature.

We first determined the location of the c-di-GMP peak by the internal standard method. It can be seen from the **FIGURE S5** that due to the interference of the side peaks, the bacterial lysate cultured in shaking state hardly sees the c-di-GMP peak, and the lysate cultured in static state can see a very small c-di-GMP peak, which is consistent with the fact that the c-di-GMP content in static state is higher than in shaking state. Moreover, by comparing with the c-di-GMP standard addition spectrum, it could be inferred that the c-di-GMP content in the lysate is less than 1 micromole, indicating that our method can effectively distinguish the low c-di-GMP levels between the two culture states.

(4) Calculation of fluorescence quantum yields

Fluorescence quantum yields (relative values) of samples were calculated according to the following expression:

$$\Phi_S = \frac{F_S}{F_R} \times \frac{(1 - 10^{-A_R})}{(1 - 10^{-A_S})} \times \frac{\eta_S^2}{\eta_R^2} \times \Phi_R$$

where the subscripts R and S respectively refer to the reference (Rhodamine B) and the sample, Φ is the fluorescence quantum yield, and the quantum yield of Rhodamine B in aqueous solution is 0.31, so $\Phi_R$is equal to 0.31, F is the integrated fluorescence intensity under fluorescence emission spectrum, A is the absorbance at the excitation wavelength and η is the refractive index of the solvent. Because the water and the buffer used in the experiment have similar refractive indices, the calculation formula can be abbreviated as:

$$\Phi_S = \frac{F_S}{F_R} \times \frac{(1 - 10^{-A_R})}{(1 - 10^{-A_S})} \times \Phi_R$$

Fluorescence integrated intensity and absorbance data are respectively obtained on PerkinElmer-LS 55 Fluorescence Spectrometer and BioTek-Epoch2 microplate reader. The calculation results are as follows:

TABLE S1. Changes of fluorescence quantum yield after adding 10 μM of c-di-GMP. Reference sample is Rhodamine B (*Φ*= 0.31, in aqueous).

**Sample**	**Ex (λmax/nm)**	**Em (λmax/nm)**	**Abs (λmax/nm)**	***Φ***
**A18**	485	546	490	0.011
**A18**+10μM c-di-GMP	485	546	490	0.211
